# pH Measurement at Elevated Temperature with Vessel Gate and Oxygen-Terminated Diamond Solution Gate Field Effect Transistors

**DOI:** 10.3390/s22051807

**Published:** 2022-02-25

**Authors:** Shuto Kawaguchi, Reona Nomoto, Hirotaka Sato, Teruaki Takarada, Yu Hao Chang, Hiroshi Kawarada

**Affiliations:** 1 Graduate School of Science and Engineering, Waseda University, 3-4-1 Okubo, Shinjuku, Tokyo 169-8555, Japan; leonardo-nmt@toki.waseda.jp (R.N.); hiro-success@suou.waseda.jp (H.S.); teruaki_mrr@moegi.waseda.jp (T.T.); yu-hao.chang@ruri.waseda.jp (Y.H.C.); 2 The Kagami Memorial Laboratory for Materials Science and Technology, Waseda University, 2-8-26 Nishiwaseda, Shinjuku, Tokyo 169-0051, Japan

**Keywords:** boron-doped diamond, pH insensitive, stainless-steel vessel, diamond solution gate field effect transistor, high temperature

## Abstract

Diamond has many appealing properties, including biocompatibility, ease of surface modification, and chemical–physical stability. In this study, the temperature dependence of the pH-sensitivity of a oxygen-terminated boron-doped diamond solution gate FET (C-O BDD SGFET) is reported. The C-O BDD SGFET operated in an electrolyte solution at 95 °C. At 80 °C, the pH sensitivity of C-O BDD SGFET dropped to 4.27 mV/pH. As a result, we succeeded in developing a highly sensitive pH sensing system at −54.6 mV/pH at 80 °C by combining it with a highly pH sensitive stainless-steel vessel.

## 1. Introduction

Measuring pH is required in a variety of industries, including the food manufacturing, medical, and agriculture industries. Various types of pH sensors have been developed to meet these needs. The most widely used of these are glass electrode pH sensors, because of their long-term stability and good reproducibility. However, conventional glass electrode pH sensors have the risks of leakage of the KCl internal solution or breakage of the glass electrode. To prevent these risks, glass electrode pH sensors have not been applied in the food industry, and all-solid-state pH sensing systems have received a great deal of attention. In the 1970s, ion-sensitive field-effect-transistors (ISFETs), which can act as a biosensor in the solution, were reported from Piet Bergveld [1,2]. Much research has been undertaken on silicon ISFETs, and small, highly pH-sensitive silicon ISFETs have been commercialized [3,4,5]. Following that, we have reported that boron-doped diamond solution gate field-effect-transistors (SGFETs) operated in an electrolyte solution environment [6]. Diamond SGFETs are directly immersed in solutions and the drain current is controlled by an electric double layer from a gate electrode. Diamond has many appealing properties, including biocompatibility, ease of surface modification, and chemical-physical stability, and therefore are suitable for biosensing applications [7]. Boron-doped carbon-based materials have also been widely studied, where density functional theory has been applied to analyze the electronic properties and band structure of boron-doped diamond and other materials [8,9]. Such research has facilitated the understanding of the boron-doped layers used in diamond SGFETs. It is important to control the surface density of carriers less than 10^13^ cm^−2^ to be modulated by field effect. It means that the thickness and volumetric concentration of boron is quite crucial to obtain FET characteristics.

Diamond SGFETs are also good candidates for pH-sensing applications, since the hole concentration of boron-doped diamond SGFETs varies with different ion concentrations of the solution [10]. These types of semiconductor pH sensors are very convenient, because they can be made smaller than glass electrode pH sensors. However, these semiconductor pH sensors use glass electrodes as gate electrodes, hence they were not suitable for applications in the food industry. Various methods have been tested to solve this problem, such as replacing the glass electrode with pH-insensitive reference FETs (REFETs) or quasi-reference electrodes (QRE) [2,11,12,13]. However, we utilize a stainless-steel vessel named “Vessel Gate” as the gate electrode instead of the glass electrodes. This is because SUS304 is widely used in the food industries due to advantages such as low cost and corrosion- and oxidation-resistance. Several studies have been conducted on stainless-steel pH sensor application, showing that SUS304 and SUS314 are pH-sensitive to approximately 57 mV/pH, with good response times [14,15,16,17]. Since the food industry has processes such as heat treatment at high temperatures, there is a demand for pH sensors that can measure even at high temperatures. 

The temperature characteristics of silicon ISFETs remain difficult, and the temperature characteristics of other semiconductor pH sensors have only been partially studied. AlGaN/GaN heterostructure pH sensors can operate at temperatures as high as 80 °C, and their pH sensitivity has been found to be stable and highly sensitive at room temperature [18]. The AlGaN/GaN heterostructure pH sensor also shows a decrease in current value as the temperature is increased. Commercially available glass-electrode pH sensors are stable and highly sensitive at room temperature, but can fail at temperatures near 100 °C [19,20,21,22]. However, since these pH measurements use glass electrodes, they may not be applicable to high-temperature measurement in the food industry. Furthermore, the temperature characteristics of diamond SGFETs have not been investigated. In this study, we investigated the temperature characteristics of oxygen-terminated (C-O) boron-doped diamond (BDD) SGFETs and their ability to operate at high temperatures in a system combined with a vessel gate. 

## 2. Materials and Methods

### 2.1. Fabrication Process and pH Measurement Systems

**Figure 1:**
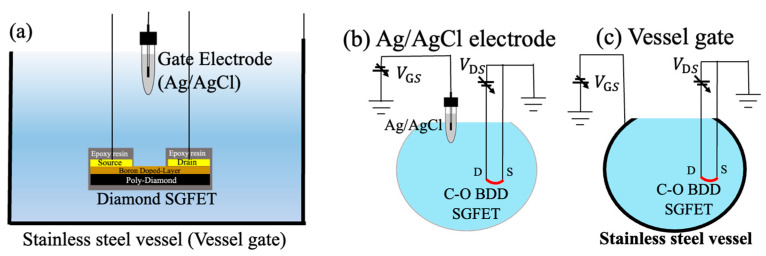
(**a**)The cross-sectional view of C-O BDD SGFETs in an electrolyte solution with Ag/AgCl electrode and vessel gate. The measurement schematic images: (**b**) Ag/AgCl electrode and (**c**) vessel gate. In the case of (**b**) Ag/AgCl electrode, the gate voltage is applied between the tip of the electrode and the FET channel which is the sensing surface. In the case of (**c**) vessel gate, the entire stainless-steel surface also becomes the sensing surface.

Two black polycrystalline diamond substrates with dimensions of 5 × 5 mm^2^ were used in this study [23,24]. One was used to measure the temperature characteristics of I_DS_-V_GS_, and the other to measure the temperature dependence of pH sensitivity. Both substrates were made in the same way. Boron-doped layer was deposited on the polycrystalline diamond using a microwave plasma chemical vapor deposition (CVD) (Arios Inc., Tokyo, Japan). The CVD conditions named in the previous paper were used [25]. The surface was cleaned in a mixture of nitric acid (HNO_3_) and sulfuric acid (H_2_SO_4_) at 200 °C for 30 min before the surface of polycrystalline diamond substrates was fully hydrogen-terminated (C-H) with microwave-enhanced plasma CVD (Astex: AX5010). Then, gold source and drain electrodes were deposited on the C-H diamond surface, except for the channel region, by using electron-beam evaporation with a thickness of 100 nm. The channel length L and width W were 200 μm and 5 mm. To connect the wires to the gold electrodes, conductive epoxy resin was used, and then all the conductive parts were covered with insulating epoxy resin. Oxygen termination (C-O) on the channel region of the diamond surface was carried out by a plasma reactor in an oxygen atmosphere. The cross-sectional diagram of C-O BDD SGFET is shown in Figure 1a. Two types of gate electrodes, an Ag/AgCl glass electrode and a vessel gate, were utilized as shown in Figure 1b,c.

### 2.2. How to Measure pH Sensitivity

The pH sensitivity was measured in the Carmody buffer solutions prepared in the same way as in the previous paper [25]. The pH of the solution was adjusted to pH 2, 4, 6, 8, 10, and 12 at room temperature with a digital pH meter (Yokogawa Electric Corp., Tokyo, Japan), and the pH was monitored at random times during high-temperature experiments. Since the pH value gradually shifts during the measurement at high temperature, the actual value was used to calculate the pH sensitivity. To raise the temperature of the solution to the operation temperature, the beaker and stainless-steel vessel were heated on a digital hot plate (As One: HP-2SA) and removed from the hot plate during measurement. The pH sensitivity was calculated by calculating the V_GS_ with the same drain current from the measurement results of the I_DS_-V_GS_ characteristic.

## 3. Results and Discussion

### 3.1. Temperature Dependence of I_DS_-V_DS_ Characteristics of C-O BDD SGFET

**Figure 2:**
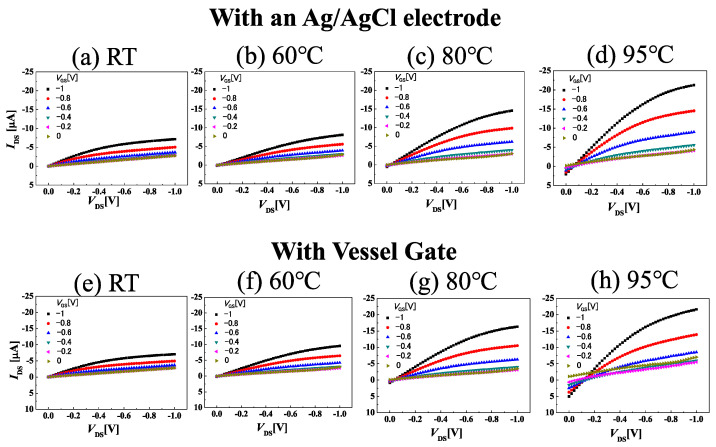
*I*_DS_-*V*_DS_ characteristics at room temperature to 95 °C when using two types of gate electrodes. (**a**–**e**) and (**f**–**h**) use an Ag/AgCl electrode and vessel gate, respectively.

Figure 2a–j show drain-source I_DS_ drain-source voltage V_DS_ characteristics at room temperature, 60, 80, and 95 °C when using an Ag/AgCl electrode and vessel gate, respectively. As the temperature increased, the drain current increased and the transconductance increased, and the gate leakage current increased at low drain-source voltage. The transconductance levels were 11, 13, 24, 34 μS for Figure 2a,d and 11, 16, 29, 39 μS for Figure 2e–h, respectively. The AlGaN/GaN heterostructure pH sensor had a decrease in drain current as the temperature increased, but the C-O BDD SGFET was interesting, because the drain current increased as the temperature increased [18]. In the case of an Ag/AgCl electrode, the transconductance was three times higher than room temperature at 95 °C. These results indicate the boron in the boron-doped layer was activated with increasing temperature. The operating mechanism of the BDD SGFETs can be thought of in the same way as the junction FET. This is because the thickness of the depletion layer controls the current in the channel. Therefore, the channel conductance of junction FET can be applied to SGFET as shown below.

(1) $$g_{channel}=\frac{e\mu_{p}N_{A}Zh}{L}$$

Here, *e* and *μ_p_* is elementary charge and hall mobility, *N_A_* is accepter concentration, *Z* is gate width, and *h* is boron-doped depth. The transconductance of BDD SGFET is related to channel conductance in junction FET, however, this formula assumes that the acceptor is completely ionized [26]. In the case of BDD SGFETs, all boron is not ionized, so *N_A_* is replaced with hole concentration *p* expressed as

(2) $$p=\left(\frac{N_{A}-N_{D}}{N_{D}}\right)\left[\frac{M_{V}}{g_{d}}\right]exp\left(-\frac{E_{i}}{k_{B}T}\right)$$

where *N_D_* is the compensating donor concentration, *M_v_* is the effective density of states of the valence band, *g_d_* is the acceptor degeneracy, *E_i_* is the ionization energy of the acceptor, *k_B_* is the Boltzmann constant, and *T* is the temperature [26]. This indicates that hole concentration *p* depends on the exponential term, which has a temperature parameter, and hence the channel conductance of BDD SGFET increases with increasing temperature. Here, since the channel conductance was tripled at 95 °C, it can be expected that the temperature-dependent exponential term was tripled compared to that at room temperature. The corresponding activation energy *Ei* is 0.15 eV between room temperature and 95 °C. The activation energy of boron decreases from 0.37 eV as the concentration of boron increases [27]. When the boron concentration is 6–8 × 10^19^ atoms cm^−3^, the activation energy of boron was reduced to 0.15 eV, which is consistent with the Pearson–Bardeen curve applied in diamond by Lagrange et al. [26]. It was 2.5 times higher than that of the exponential term at room temperature as shown in Table 1. 

**Figure 3:**
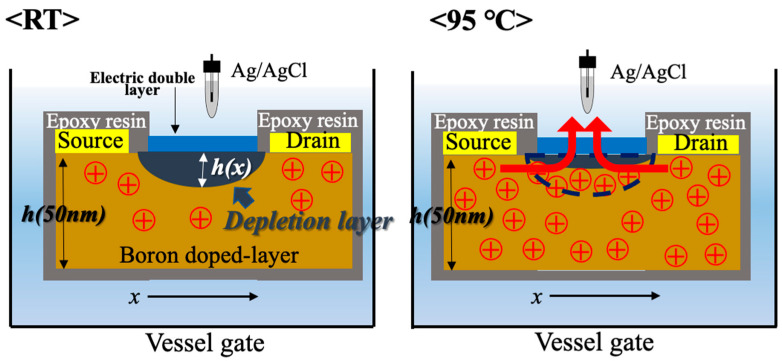
The state of the depletion layer of the C-O BDD SGFETs when the drain-source voltage is low and the gate leakage current is flowing. At 95 °C, when the drain-source voltage is small, the depletion layer is not sufficiently formed and a gate leakage current is generated.

In addition to the increase in channel conductance, there was also an increase in gate leakage current with increasing temperature at low drain-source voltage. The gate leakage current is thought to be due to tunneling that occurs when the insulating film becomes thin. In the case of the BDD SGFETs, the thickness of depletion layer *h(x)* is expressed as:

(3) $$h\left(x\right)={\left[\frac{2\varepsilon \left(-V\left(x\right)-V_{bi}+V_{G}\right)}{eN_{A}^{-}}\right]}^{\frac{1}{2}}$$

where ε is electric constant, *V(x)* is the channel potential, *V_bi_* is the built-in voltage, *V_G_* is the gate voltage, and $N_{A}^{-}$ is the concentration of activated boron. This formula suggests that the thickness of the depletion layer decreases as boron in the channel is activated. The amount of boron that was ionized increased as the temperature rises, and the boron that was in the depletion layer was ionized, which made the depletion layer thinner, as shown Figure 3. However, this gate leakage current distinctively occurred at a higher forward bias of this diode. The BDD SGFET used in this study had a gate length of 200 μm, so by passing this to about 5 μm, the gate leakage current could be increased, as shown in this reference [28]. The results showing that BDD SGFETs operated in harsh environments at 95 °C indicate that BDD SGFETs are great candidates for high-temperature pH measurement.

### 3.2. Temperature Dependence of pH Sensitivity of C-O BDD SGFET

**Figure 4:**
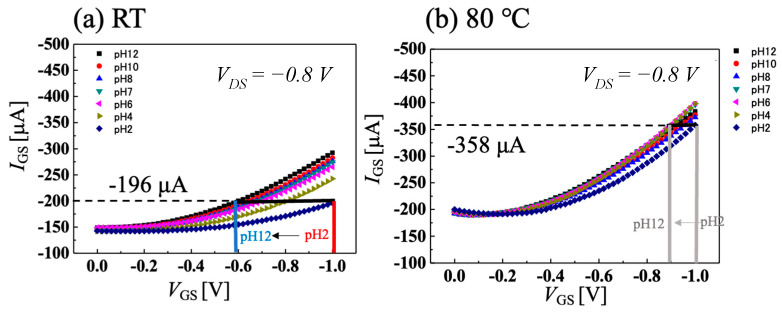
pH measurement using Ag/AgCl electrode at V_DS_ = −0.8 V when solution temperature is (**a**) room temperature and (**b**) 80 °C.

**Figure 5:**
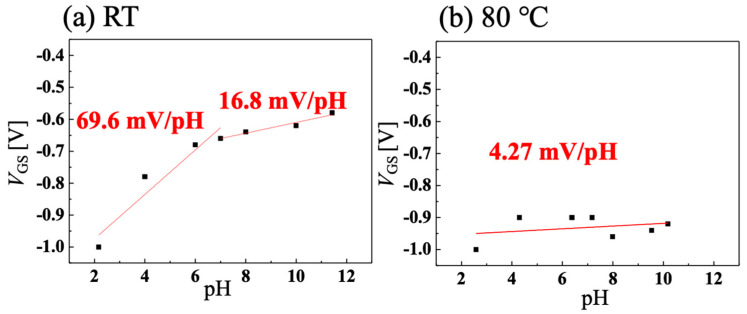
pH sensitivity calculated from Figure 4a,b when solution temperature is (**a**) room temperature and (**b**) 80 °C.

Figure 4a,b show the temperature dependence of *I-V* characteristics of C-O BDD SGFETs at room temperature and 80 °C when using Ag/AgCl electrode as a gate electrode, and Figure 5a,b show the temperature dependence of pH sensitivity calculated from Figure 4a,b, respectively. Our FET operates at 95 °C, however, the highest temperature of pH-sensing measurement in our facility was 80 °C. The C-O BDD SGFETs showed a high pH sensitivity of 69.6 mV/pH in the acidic region and a medium sensitivity of 16.8 mV/pH in the alkaline region at room temperature. By contrast, the C-O BDD SGFETs showed low pH sensitivity of 4.27 mV/pH at 80 °C. As a result, we have succeeded in manufacturing a pH-insensitive semiconductor pH sensor, which is difficult to manufacture, in a high-temperature solution. The reason why the pH sensitivity of C-O BDD SGFETs becomes low at high temperature is that the boron acceptor is activated, and holes increase at high temperature. In other words, since the amount of boron activated increases, the effect of changes in drain current caused by surface-adsorbed ions due to changes in pH is reduced, and the sensitivity to pH is reduced. If adsorbents are attached to the channel surface of the C-O BDD SGFET during the measurement in a hot solution, the pH sensitivity after high-temperature measurement may change due to the influence of the adsorbents. Figure S1a,b (Supplementary Material) show the *I-V* characteristics and pH sensitivity of the C-O BDD SGFET with Ag/AgCl at room temperature after pH sensitivity measurement between room temperature and 80 °C. The C-O BDD SGFET was highly pH-sensitive in the acidic region and medium pH-sensitive in the alkaline region, as with the initial room temperature measurement.

### 3.3. pH Sensing System at High Temperature with C-O BDD SGFET and Vessel Gate

**Figure 6:**
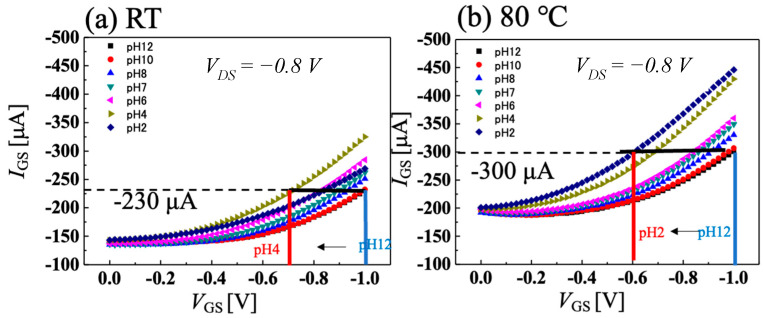
pH measurement using vessel gate at V_DS_ = −0.8 V when solution temperature is (**a**) room temperature and (**b**) 80 °C.

**Figure 7:**
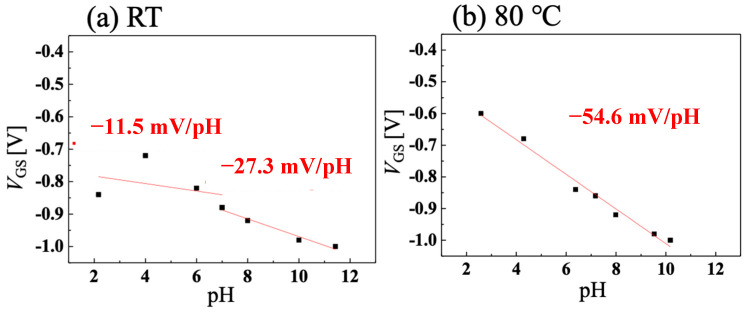
pH sensitivity calculated from Figure 6a,b when solution temperature is (**a**) room temperature and (**b**) 80 °C.

Figure 6a,b show the results of the *I-V* characteristics combined with a highly pH-sensitive vessel gate for application in the food industry at room temperature and 80 °C. Figure 7a,b show the temperature dependence of pH sensitivity calculated from Figure 6a,b, respectively. The pH-sensitivity measurement was performed when the vessel gate and C-O BDD SGFET were combined without Ag/AgCl electrode, as shown in Figure 1c. Focusing on room temperature, the acidic region, which was highly sensitive at 69.6 mV/pH with the Ag/AgCl electrode, became pH insensitive at −11.5 mV/pH. In addition, the alkaline region, which was medium-sensitive at 16.8 mV/pH with the Ag/AgCl electrode, became −27.3 mV/pH. The positive and negative pH sensitivities changed in the opposite direction, because, in a sensing circuit, the direction of the surface dipole on the FET channel is opposite to that of the vessel surface. As a result, the ion effects on the two electric double layer capacitors on the FET channel and vessel surface were opposite each other. The analytical explanation for the opposite sensitivity has been described in another article in detail [29]. These results suggest that the vessel gate is highly sensitive in the opposite direction from which it reverses the sensitivity of the C-O BDD SGFET. In the acidic region at room temperature, if both pH sensitivities were superpositioned opposite to each other, the resulting pH sensitivity was low. The combination of the C-O BDD SGFET at 80 °C and the vessel gate creates a case in which the pH-insensitive material is superpositioned on a highly pH-sensitive material. Using a highly pH-sensitive vessel gate and pH-insensitive SGFETs, it was measured at −54.6 mV/pH. This result indicates that a pH-insensitive FET is best combined with a pH-sensitive vessel gate. Since the Nernst response at 80 °C is 70.1 mV/pH, we were able to realize a pH sensor that has a sensitivity of 77.9% and can be applied in the food industry where high-temperature treatment is needed.

## 4. Conclusions

The C-O BDD SGFETs were able to operate in a harsh environment of 95 °C. The transconductance increased and the gate leakage current increased as the temperature of the solution increased because of the activated boron. The C-O BDD SGFETs were highly pH-sensitive in the acidic region and medium pH-sensitive in the alkaline region at room temperature, however, they became pH insensitive to 4.27 mV/pH at high temperatures. By combining the pH-sensitive vessel gate and the pH-insensitive C-O BDD SGFETs, a system with a high pH sensitivity of −54.6 mV/pH was realized at 80 °C.

## Figures and Tables

**Table 1:** Comparison of exponential term values when the activation energy of boron is 0.15 eV. As a result, it is considered that the channel conductance tripled when the activation energy of boron was 0.15 eV.

	$exp\left(\frac{E_{i}}{k_{B}T}\right)$		
Activation energy of boron [eV]	298 [K]	368 [K]	368 [K]/298 [K]
0.15	0.0029	0.0088	3.0

## Supplementary Materials

The following supporting information can be downloaded at: https://www.mdpi.com/article/10.3390/s22051807/s1, Figure S1: (a) I-V characteristics and (b) pH sensitivity of C-O BDD SGFET with Ag/AgCl at room temperature after pH sensitivity measurement at room temperature and 80 ℃. The pH sensitivity was the same as room temperature, high in the acidic region, and medium sensitive in the alkaline region.

## References

[1] Bergveld P. (1970). Development of an ion-sensitive solid-state device for neurophysiological measurements. IEEE Trans. Biomed. Eng..

[2] Bergveld P. (1972). Development, Operation, and Application of the Ion-Sensitive Field-Effect Transistor as a Tool for Electrophysiology. IEEE Trans. Biomed. Eng..

[3] Matsuo T., Wise K.D. (1974). An Integrated Field-Effect Electrode for Biopotential Recording. IEEE Trans. Biomed. Eng..

[4] Bausells J., Carrabina J., Errachid A., Merlos A. (1999). Ion-sensitive field-effect transistors fabricated in a commercial CMOS technology. Sens. Actuators B Chem..

[5] Matsuo T., Nakajima H. (1984). Characteristics of reference electrodes using a polymer gate ISFET. Sens. Actuators.

[6] Kawarada H., Araki Y., Sakai T., Ogawa T., Umezawa H. (2001). Electrolyte-Solution-Gate FETs Using Diamond Surface for Biocompatible Ion Sensors. Phys. Status Solidi A.

[7] Kawarada H., Ruslinda A.R. (2011). Diamond electrolyte solution gate FETs for DNA and protein sensors using DNA/RNA aptamers. Phys. Status Solidi A.

[8] Hellgren N., Berlind T., Gueorguiev G.K., Johansson M.P., Stafström S., Hultman L. (2004). Fullerene-like BCN thin films: A computational and experimental study. Mater. Sci. Eng. B.

[9] Medeiros P.V., Gueorguiev G.K., Stafström S. (2015). Bonding, charge rearrangement and interface dipoles of benzene, graphene, and PAH molecules on Au (1 1 1) and Cu (1 1 1). Carbon.

[10] Song K., Nakamura Y., Sasaki Y., Degawa M., Yang J., Kawarada H. (2006). pH-sensitive diamond field-effect transistors (FETs) with directly aminated channel surface. Anal. Chim. Acta.

[11] Salm E., Zhong Y., Reddy B., Duarte-Guevara C., Swaminathan V., Liu Y., Bashir R. (2014). Electrical detection of nucleic acid amplification using an on-chip quasi-reference electrode and a PVC REFET. Anal. Chem..

[12] Chang K., Chang C., Chao K., Chen J. (2010). Development of FET-type reference electrodes for pH-ISFET applications. J. Electrochem. Soc..

[13] Bergveld P., Van Den Berg A., Van Der Wal P.D., Skowronska-Ptasinska M., Sudhölter E., Reinhoudt D.N. (1989). How electrical and chemical requirements for REFETs may coincide. Sens. Actuators.

[14] Hashimoto T., Nakade K., Ishihara A., Nishio Y. (2021). Development of Ag and Ag alloys-precipitated Ag_2_O-TeO_2_ glass and Ag2O-TeO2 glass/stainless steel reference electrodes for pH sensors. Sens. Actuators B Chem..

[15] Hashimoto T., Kitabayashi H., Ito K., Nasu H., Ishihara A., Nishio Y. (2019). Effect of heat-treatment on the pH sensitivity of stainless-steel electrodes as pH sensors. Heliyon.

[16] Nomura K., Ujihira Y. (1990). Analysis of oxide layers on stainless steel (304, and 316) by conversion electron Mössbauer spectrometry. J. Mater. Sci..

[17] Nomura K., Ujihira Y. (1988). Response of oxide films on stainless steel as a pH sensor. Anal. Chem..

[18] Niigata K., Narano K., Maeda Y., Ao J. (2014). Temperature dependence of sensing characteristics of a pH sensor fabricated on AlGaN/GaN heterostructure. Jpn. J. Appl. Phys..

[19] Manjakkal L., Szwagierczak D., Dahiya R. (2020). Metal oxides based electrochemical pH sensors: Current progress and future perspectives. Prog. Mater. Sci..

[20] Kinlen P.J., Heider J.E., Hubbard D.E. (1994). A solid-state pH sensor based on a Nafion-coated iridium oxide indicator electrode and a polymer-based silver chloride reference electrode. Sens. Actuators B Chem..

[21] Clark Westcott C. (1973). pH Measurements.

[22] Bates R.G. (1973). Determination of pH. Theory and Practice.

[23] Syamsul M., Kitabayashi Y., Matsumura D., Saito T., Shintani Y., Kawarada H. (2016). High voltage breakdown (1.8 kV) of hydrogenated black diamond field effect transistor. Appl. Phys. Lett..

[24] Williams O.A., Jackman R.B., Nebel C., Foord J.S. (2002). Black diamond: A new material for active electronic devices. Diam. Relat. Mater..

[25] Chang Y.H., Iyama Y., Tadenuma K., Kawaguchi S., Takarada T., Falina S., Syamsul M., Kawarada H. (2021). Over 59 mV pH− 1 Sensitivity with Fluorocarbon Thin Film via Fluorine Termination for pH Sensing Using Boron-Doped Diamond Solution-Gate Field-Effect Transistors. Phys. Status Solidi A.

[26] Barjon J., Chikoidze E., Jomard F., Dumont Y., Pinault-Thaury M., Issaoui R., Brinza O., Achard J., Silva F. (2012). Homoepitaxial boron-doped diamond with very low compensation. Phys. Status Solidi A.

[27] Lagrange J., Deneuville A., Gheeraert E. (1998). Activation energy in low compensated homoepitaxial boron-doped diamond films. Diam. Relat. Mater..

[28] Iwai H. (1995). Momose, H. Miniaturization and performance limits of silicon devices. Appl. Phys..

[29] Chang Y.H. (2021). New All-solid-state pH Sensing System Utilizing Diamond Solution Gate Field-Effect Transistors and Stainless Steel Vessel Gate. Sens. Actuators B Chem..

