# Supplementary material for: pH Measurement at Elevated Temperature with Vessel Gate and Oxygen-Terminated Diamond Solution Gate Field Effect Transistors
Source: Sensors (Basel). 2022 Feb 25;22(5):1807. doi: 10.3390/s22051807 (PMC8915005; doi:10.3390/s22051807)
Supplement: Supplementary file 1 [file sensors-22-01807-s001.zip › sensors-1583743-supplementary.pdf]

Supplementary material for

pH Measurement at Elevated Temperature with Vessel Gate and Oxygen-terminated Diamond Solution Gate Field Effect Transistor

Shuto Kawaguchi[1]*, Reona Nomoto[1], Teruaki Takarada[1], Hirotaka Sato[1], Yu Hao Chang[1] and Hiroshi Kawarada[1,2,] **

[1]Graduate School of Science and Engineering, Waseda University, 3-4-1 Okubo, Shinjuku, Tokyo 169-8555, Japan

[2]The Kagami Memorial Laboratory for Materials Science and Technology, Waseda University, 2-8-26 Nishiwaseda, Shinjuku, Tokyo 169-0051, Japan

Email: syuto-1227@fuji.waseda.jp

Changes in pH sensitivity after high temperature measurement

The pH sensitivity was measured again after the 80 °C measurement in order to investigate whether the pH sensitivity was affected by the surface adsorbate. The pH sensitivity was the same as before the measurement at high temperature, with high pH sensitivity in the acidic region and medium pH sensitivity in the alkaline region. This suggest that the pH insensitivity in high temperature measurements was not due to the effect of surface adsorbents, but to the effect of boron activation, which changed the pH sensitivity.

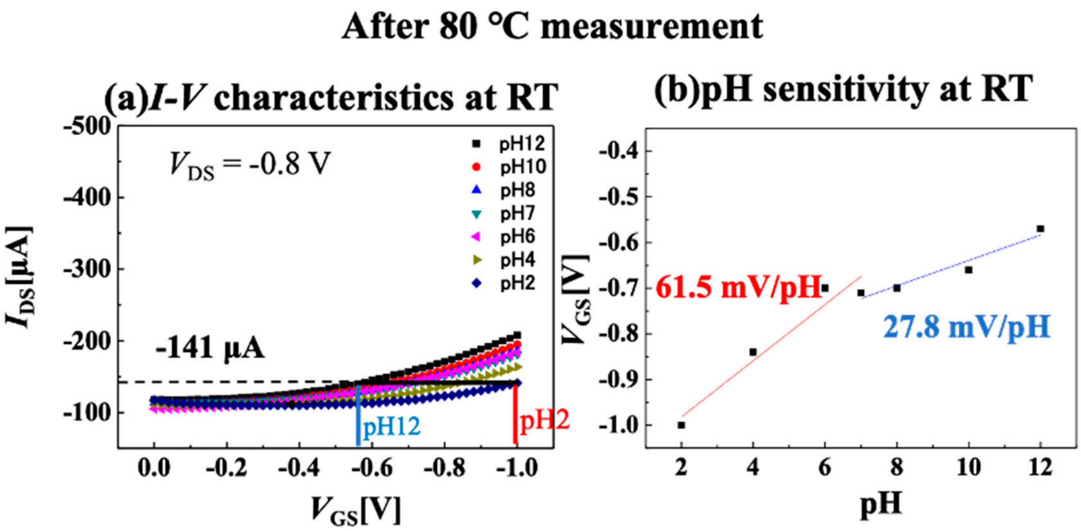

Figure S1. (a)I-V characteristics and (b)pH sensitivity of C-O BDD SGFET with Ag/AgCl at room temperature after pH sensitivity measurement at room temperature and 80 °C. The pH sensitivity was the same as room temperature, high in the acidic region, and medium sensitive in the alkaline region.
